# Isolation and characterization of ZZ1, a novel lytic phage that infects *Acinetobacter baumannii* clinical isolates

**DOI:** 10.1186/1471-2180-12-156

**Published:** 2012-07-28

**Authors:** Jing Jin, Zhen-Jiang Li, Shu-Wei Wang, Shan-Mei Wang, De-Hai Huang, Ya-Hui Li, Yun-Yun Ma, Jin Wang, Fang Liu, Xiang-Dong Chen, Guang-Xing Li, Xiao-Ting Wang, Zhong-Quan Wang, Guo-Qiang Zhao

**Affiliations:** 1 Department of Pathogen Biology, Basic Medical College of Zhengzhou University, Kexue Road # 100, Zhengzhou, 450001, P. R. China; 2 Department of Pathogen Biology and Immunology, Henan Medical College for Staff and Workers, Zhengzhou, China; 3 State Key Laboratory of Virology, Wuhan University, Wuhan, China; 4 Clinical Laboratory, Henan Province People’s Hospital, Zhengzhou, China

**Keywords:** *Acinetobacter baumannii *, Bacteriophage, Characterization

## Abstract

**Background:**

*Acinetobacter baumannii*, a significant nosocomial pathogen, has evolved resistance to almost all conventional antimicrobial drugs. Bacteriophage therapy is a potential alternative treatment for multidrug-resistant bacterial infections. In this study, one lytic bacteriophage, ZZ1, which infects *A. baumannii* and has a broad host range, was selected for characterization.

**Results:**

Phage ZZ1 and 3 of its natural hosts, *A. baumanni* clinical isolates AB09V, AB0902, and AB0901, are described in this study. The 3 strains have different sensitivities to ZZ1, but they have the same sensitivity to antibiotics. They are resistant to almost all of the antibiotics tested, except for polymyxin. Several aspects of the life cycle of ZZ1 were investigated using the sensitive strain AB09V under optimal growth conditions. ZZ1 is highly infectious with a short latent period (9 min) and a large burst size (200 PFU/cell). It exhibited the most powerful antibacterial activity at temperatures ranging from 35°C to 39°C. Moreover, when ZZ1 alone was incubated at different pHs and different temperatures, the phage was stable over a wide pH range (4 to 9) and at extreme temperatures (between 50°C and 60°C). ZZ1 possesses a 100-nm icosahedral head containing double-stranded DNA with a total length of 166,682 bp and a 120-nm long contractile tail. Morphologically, it could be classified as a member of the *Myoviridae* family and the *Caudovirales* order. Bioinformatic analysis of the phage whole genome sequence further suggested that ZZ1 was more likely to be a new member of the *Myoviridae* phages. Most of the predicted ORFs of the phage were similar to the predicted ORFs from other *Acinetobacter* phages.

**Conclusion:**

The phage ZZ1 has a relatively broad lytic spectrum, high pH stability, strong heat resistance, and efficient antibacterial potential at body temperature. These characteristics greatly increase the utility of this phage as an antibacterial agent; thus, it should be further investigated.

## Background

Bacteriophage therapy is one of the emerging methods used to overcome bacterial infections [1-3]. Bacteriophages are viruses that infect and kill bacteria. Theoretically, phages have several advantages over antibiotics. They are highly specific and very effectively lyse targeted pathogenic bacteria. They are safe because they have no activity against animal or plant cells. Phages are ubiquitous, so isolation of new phages is a relatively rapid process that can frequently be accomplished in days or weeks. The use of phages as therapeutic agents was initiated in 1919, 3 years after their discovery, for the treatment of bacillary dysentery and continued until the 1940s. Over this time period, phages were used to treat a variety of infectious diseases [4]. However, with the advent of antibiotics, commercial production of therapeutic phages ceased in most of the Western world [5]. Currently, there is renewed interest in phage research and the applications of bacteriophages as potentially powerful antibacterial agents due to the emergence of drug-resistant pathogens and the dearth of new antibiotics. Several studies have shown that bacteriophages can be used successfully for therapeutic purposes, both in humans and animals [6-9]. However, more research is required before clinical use can be re-initiated. Before using a phage for therapeutic purposes, the isolation of lytic phages and characterization of the phage are essential.

In this study, clinical isolates of *Acinetobacter baumannii* were collected and used as indicator hosts to screen phages from water samples. *A. baumannii* mostly infects debilitated patients in intensive care units and is associated with high mortality rates [10,11]. Since its discovery, *A. baumannii* has become resistant to many common antibiotics [12]. The increasing prevalence of multidrug- and pandrug-resistant *A. baumannii* strains in clinics has rendered it one of the most important nosocomial pathogens [12-15]. Fortunately, lytic phages specific to *A. baumannii* might provide an alternative to antibiotics for the prevention and treatment of infections caused by this bacterium. However, to the best of our knowledge, very few detailed characterizations of *A. baumannii* phages have been published [16,17]. This paper describes the isolation and characterization of a novel virulent phage, ZZ1, that infects *A. baumannii* clinical isolates.

## Results

### Isolation of ZZ1 and its morphology

**Figure 1:**
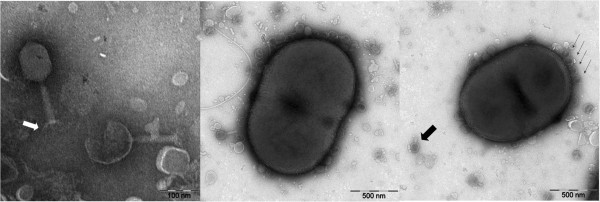
**Electron micrographs of ZZ1 and infected *A. baumannii* AB09V.** A mixture of ZZ1 phages and *A. baumannii* AB09V cells was negatively stained. The phage ZZ1 contained a baseplate with fibers (indicated by the white arrow) and an icosahedral head with a contractile tail (indicated by the large black arrow), which allowed for its inclusion in the *Myoviridae* family of the order *Caudovirales*. Intact phages had a head filled with DNA, and ghost particles (indicated by the small black arrows) had an empty head, showing that ejection of DNA from the phage head had taken place within 5 min.

Twenty-three *A. baumannii* clinical isolates were screened for phage present in a sample of fishpond water. Among these, only the strain AB09V could serve as an indicator for ZZ1 in the initial screening. This phage formed clear plaques of approximately 1-2 mm in diameter on AB09V lawns. AB09V was thus used to propagate, purify and characterize the phage. As shown in Figure 1, the phage ZZ1 has a 100-nm icosahedral head and a 120-nm long contractile tail. Morphologically, phage ZZ1 can be tentatively classified as a member of the *Myoviridae* family in the order of *Caudovirales*. Most of the input phages rapidly adsorbed to AB09V cells. Appearance of ghost particles 5 min after mixing phages with bacteria suggested that ejection of DNA from the phage head occurred rapidly.

### Host range of ZZ1 and identification of bacterial strains

**Figure 2:**
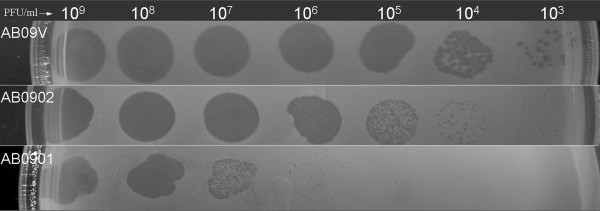
**Antibacterial activity of phage ZZ1 against three *A.*  *baumannii strains. * **Serial 10-fold dilutions of phage ZZ1 were spotted onto lawns of strains AB09V, AB0901, and AB0902 in 0.7% agar nutrient broth at 37°C. AB09V was used as the indicator for determination of the phage titer.

Two additional natural bacterial hosts, AB0901 and AB0902, were found when the other 22 of the 23 *A. baumannii* clinical isolates were used to investigate the host range of ZZ1 by spot test. This test used a higher concentration of phage (10^8^ PFU/ml) than the original screen. Interestingly, some differences were observed in the ability of the phage to lyse the 3 bacterial hosts (AB09V, AB0901, and AB0902). For example, as shown Figure 2, ZZ1 was capable of forming transparent areas on lawns of the strains AB09V, AB0901, and AB0902. However, the minimum phage concentrations required to form clear spots on each lawn were different: AB09V required 10^5^ PFU/ml, AB0902 required 10^6^ PFU/ml, and AB0901 required 10^8^ PFU/ml. The values suggest that under the same culture conditions, the antibacterial activity of ZZ1 was highest in strain AB09V, followed by AB0902 and then AB0901. There might be natural resistance mechanisms in AB0901 and AB0902; thus, the strain AB09V is likely the most sensitive indicator of the phage titer of the 3 strains and is the best host for phage propagation.

The 3 strains that can be infected by ZZ1 were selected for further detailed study. They were resistant to all of the antibiotics tested except polymyxin (amikacin, gentamicin, imipenem, meropenem, cefazolin, ceftazidime, cefotaxime, cefepime, aztreonam, ampicillin, piperacillin, amoxicillin/clavulanic acid, ampicillin/sulbactam, piperacillin/tazobactam, sulfanilamide, sulfamethoxazole and trimethoprim, ciprofloxacin, levofloxacin, and tetracycline). Partial 16 S rRNA genes of the 3 strains were sequenced and deposited in GenBank under the accession numbers [GenBank: JF313142] (AB09V), [GenBank: JF313143] (AB0901), and [GenBank: JF313144] (AB0902). Nucleotide blast analysis further confirmed that the three strains were *A. baumannii*.

### Stability investigation

**Figure 3:**
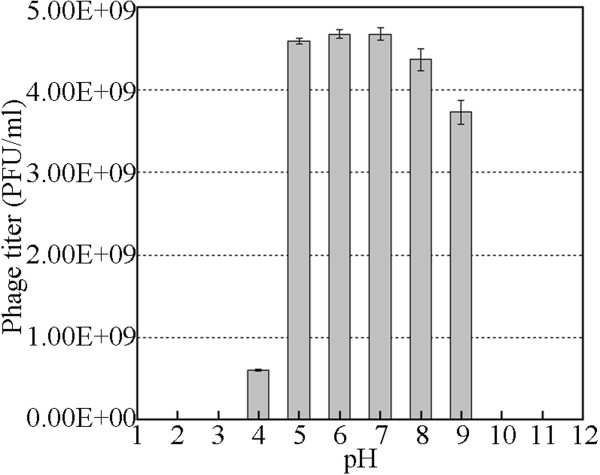
**ZZ1 stability test based on pH.** The phage ZZ1 was incubated at different pH values for one hour before determination of the number of infectious phage particles.

**Figure 4:**
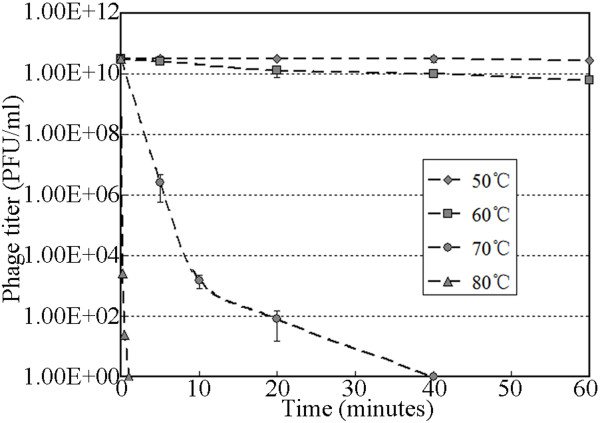
**ZZ1 heat stability test.** Samples were taken at different time intervals to determine the titer of the surviving infectious phage particles.

Temperature and pH stability are two important parameters in the storage of therapeutic phage. Thus, the stability of ZZ1 was investigated at different pHs and temperatures. As shown in Figure 3, no obvious effect on ZZ1 activity was observed after 1 h of incubation at pH levels ranging from 4 to 9. However, the phage completely lost its infectivity at pH 10 or higher and pH 3 or lower. Within 1 h of incubation at pH 4, the phage infectivity decreased by approximately 87%, and a titer of 6.0 × 10^8^ PFU/ml of active phage ZZ1 was detected at the end of the incubation. The maximum stability of the phage was observed at a pH between 6 and 7. In addition, the results of thermal stability tests shown in Figure 4 suggest that ZZ1 was relatively heat stable over 1 h at temperatures between 50°C and 60°C, and no significant loss in phage activity was observed. At 70°C, the phage titer quickly dropped, and no viral particles were detected after 40 min. Furthermore, phage activity was completely lost at 80°C within the first 1 min of incubation. The ZZ1 phage lysate retained almost 100% of its infection activity when stored at both 25°C and 4°C for several months (data not shown).

### Investigation of antimicrobial activity of ZZ1 against AB09V at different temperatures

**Figure 5:**
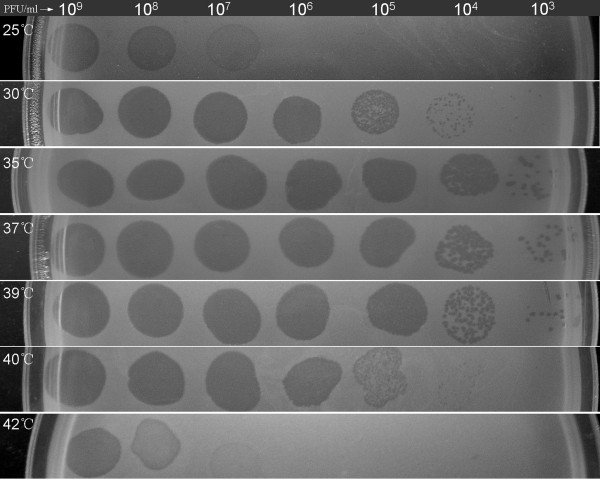
**Optimal temperature for antibacterial activity of ZZ1 against *A. baumannii* AB09V.** Serial 10-fold dilutions of phage ZZ1 were spotted onto lawns of the sensitive strain AB09V in 0.7% agar nutrient broth at different temperatures.

Optimal *A. baumannii* growth occurs over a very broad temperature range [10]. As shown in Figure 5, the AB09V lawns grew well on nutrient agar plates at temperatures ranging from 25°C to 42°C. However, the antimicrobial activity of ZZ1 is clearly influenced by temperature variations. When the plates were incubated at different temperatures, the minimum phage concentrations required to form clear spots on AB09V lawns were different: 10^5^ PFU/ml at 35°C, 37°C, and 39°C; 10^6^ PFU/ml at 30°C and 40°C; 10^8^ PFU/ml at 25°C; and 10^9^ PFU/ml at 42°C. Thus, we concluded that ZZ1 exhibits the most efficient antibacterial activity against the AB09V strain at temperatures ranging from 35°C to 39°C, which suggested that ZZ1 could exhibit powerful antibacterial activity against AB09V at body temperature.

### Phage growth attributes on AB09V

**Figure 6:**
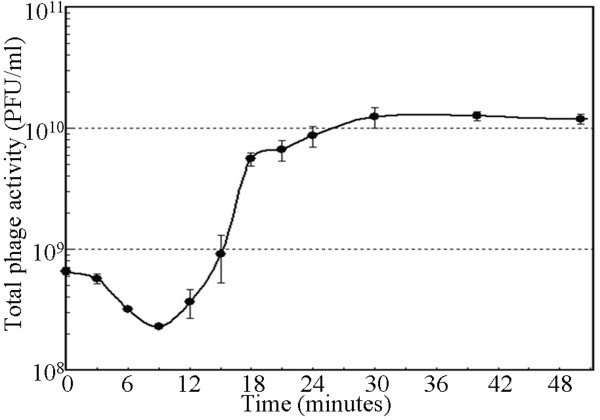
**One-step growth curve of ZZ1 on *A. baumannii* AB09V.** Phage ZZ1 was mixed with strain AB09V at an MOI of approximately 10 at 37°C (The initial ratio of phage concentration to bacterial concentration is 6.6 × 10^8^ PFU/ml: 7.0 × 10^7^ CFU/ml). Then, the total phage activity (including infected bacterial cells and free phages) was determined periodically. The decline in the concentration of total phages occurred as a result of the binding of multiple viral particles to one susceptible bacterial cell followed by a rapid increase, resulting in release of phages by lysis of the infected bacterial cells. The ZZ1 latent period was approximately 9 min, and the burst size averaged 200 PFU per infected cell.

The growth characteristics of ZZ1 on the sensitive indicator strain AB09V were characterized under optimal growth conditions. Phage ZZ1 exhibited high infection efficiency after mixing the phages and AB09V cells. We inferred that almost all of the *A. baumannii* AB09V were infected prior to the burst time of the first infected cell because the number of bacteria surviving at 9 min was less than 100 CFU/ml. Moreover, as shown in Figure 6, the total plaque count was 6.6 × 10^8^ PFU/ml at the beginning of infection (0 min), and only 2.3 × 10^8^ PFU/ml remained after 9 min. The difference (approximately 4.3 × 10^8^ PFU/ml) originated from adsorption of multiple phage particles to one susceptible bacterial cell. The decrease in the number of phages was greater than 6-fold higher than the initial number of bacterial cells (approximately 7 × 10^7^ CFU/ml). These results further confirmed that almost all of the bacterial cells could be infected within the latent period (9 min). The number of unattached phages at the end of the latent period (or prior to the burst time of the first infected cells) can be estimated as the difference between the number of the total plaque count and the initial number of bacterial cells. The calculated number of unattached phages was 1.6 × 10^8^ PFU/ml, which is negligible compared to the phage number at the end of the experiment (1.5 × 10^10^ PFU/ml). Moreover, the number of bacteria surviving at the end of the experiment is less than 50 CFU/ml, which can also be considered negligible when compared to the initial number of bacterial cells (7.0 × 10^7^ CFU/ml). Therefore, the average burst size was approximately 200 PFU/cell, which can be calculated as the ratio of the final count of phage particles to the initial count of infected bacterial cells.

### Genome analysis

The phage nucleic acid was susceptible to degradation by DNase and some restriction endonucleases (e.g., HindIII, EcoRI, and EcoRV) but unaffected by RNase. Thus, ZZ1 is a dsDNA phage (data not shown). The ZZ1 genome has a total length of 166,682 bp and a GC content of 34.3%, which is slightly lower than that described for the *A. baumannii* ATCC 17978 strain (38%, accession number NC_009085). An initial NCBI nucleotide blast analysis (blastn) of the complete genome sequence indicated that ZZ1 shares limited similarities with other known phage nucleotide sequences, which confirmed its status as a novel *Acinetobacter* phage species. The top 4 most similar sequences found were of the *Acinetobacter* phages Acj9 [GenBank: HM004124.1], Acj61 [GenBank: GU911519.1], Ac42 [GenBank: HM032710.1], and 133 [GenBank: HM114315.1]. The max scores were 4662 (50% of coverage, 89% of max ident), 4448 (45% of coverage, 87% of max ident), 2634 (34% of coverage, 94% of max ident), and 2210 (31% of coverage, 92% of max ident). The four *Acinetobacter* phages were recently deposited in GenBank and were previously annotated as T4-like phages [18]. No other *Acinetobacter* phages were hit by blastn. In addition, *Enterobacteria* phage T4 ranked tenth, and its max score was 1972 (28% of coverage, 83% of max ident), suggesting that the ZZ1 phage might be a new member of the T4-like phage family.

A sequence search using the NCBI open reading frame (ORF) finder revealed a total of 402 putative ORFs of 50 or more codons in the ZZ1 genome that have limited similarity to other known phage proteins. Among them, 118 ORFs have the highest similarity to predicted ORFs from the *Acinetobacter* phage Acj9; 47 ORFs are most similar to predicted ORFs from the *Acinetobacter* phage Acj61; 18 ORFs most closely resemble predicted ORFs from the *Acinetobacter* phage 133; and only 13 ORFs have the highest score with predicted ORFs from the *Acinetobacter* phage Ac42. In addition, of the 402 ORFs, 105 ORFs showed homology with sequences in GenBank with annotated function; 244 ORFs had matches with uncharacterized entries; and the remaining 53 ORFs had no match to sequenced genes in the database.

## Discussion

Phage therapy has been the subject of several recent reviews, and the present study reinforces the view that it is worth exploring [1,2,19]. To the best of our knowledge, the characterization of lytic phages of *A. baumannii* has rarely been studied, although Ackermann et al. [16,20] described the classification of an *A. baumannii* phage, and Soothill et al. [1,21] tested the efficacy of phage therapy for experimental *A. baumannii* infections in mice. In this study, we focused our efforts on the isolation and characterization of *A. baumannii* phages with potential for prophylactic/therapeutic use.

Phages are thought to be found wherever bacteria thrive [22]. *Acinetobacter spp*. are ubiquitous organisms that can be readily isolated from non-clinical sources, such as soil, drinking and surface waters, sewage, and a variety of different foods [10], which suggests that phages specific to *A. baumannii* might also be easily isolated from nature. Recently, 10 phages were obtained from wastewater using 125 clinical isolates of *A. baumannii* as indicator hosts [20,23]. These phages were designated AB1 to AB9 and AB11. Examination by transmission electron microscopy suggested that phages AB1-7 and AB9 belonged to the *Podoviridae* family, and phages AB8 and AB11 belonged to the *Myoviridae* family*.* Two of the 10 phages, AB1 and AB2, were further characterized at 35°C and 37°C, respectively. Based on morphology and genomic analysis, the two phages were classified as new members of the ϕKMV-like phages [20,23]. In this study, the phage ZZ1, which is specific to *A. baumannii,* was isolated from fishpond water, which further confirmed that phages specific to *A. baumannii* are waiting to be exploited as an abundant natural resource. The ability of phage ZZ1 to form clear plaques on lawns of AB09V is indicative of lytic phage, and a large burst size with a short latent period is further suggestive of the lytic nature of phage ZZ1. Morphologically, ZZ1 exhibits features similar to the *Myoviridae* family (order *Caudovirales*), which, broadly, are tailed phages with icosahedral head symmetry and contractile tail structures. Genome analysis of ZZ1 showed that it is fairly similar to four other *Acinetobacter* phages (Acj9, Acj61, Ac42, and 133). In a recent review by Petrov et al. [18], the four *Acinetobacter* phages were initially assigned to the “T4-like Viruses” genus. Each of these *Acinetobacter* phages has a unique set of ORFs that occupy ~35% of the genome. That is, each represents a different type of T4-related phage genome [18]. The genome size of the phage ZZ1 (166,682 bp) is also close to the genome size of T4-like phages. These genomes vary between ~160,000 and ~250,000 bp [18]. Therefore, the above features confirmed that the phage ZZ1 is most likely a new member of the T4-like virus family of *Acinetobacter* phages. However, according to the 2011 Virus Taxonomy List (current) from the International Committee for the Taxonomy of Viruses (http://www.ncbi.nlm.nih.gov/ICTVdb/index.htm.), only the *Acinetobacter* phage 133 can be searched and classified in the unassigned genus of the *Myoviridae* family, most likely because the phage is inadequately characterized. At the very least, the current sequence database for the *Myoviridae* phages should prove to be a rich source of genetic markers for bioprospecting and a mine of reagents for basic research and biotechnology. Our future research will focus on further detailed analysis of the whole ZZ1 genome to understand the genetic characteristics of this phage.

The main aim of this study was the isolation and characterization of a lytic bacteriophage with potential for prophylactic/therapeutic use. Therefore, the antibacterial activity of the phage against its different host cells was the focus of our research. Through the preliminary *in vitro* host range investigation, ZZ1 was found to have different antibacterial activity against 3 of its natural hosts. The antibacterial activity of ZZ1 was highest against the strain AB09V, followed by AB0902 and then AB0901, based on the minimum phage concentration required to form clear spots at 37°C. The natural resistance mechanisms of AB0901 and AB0902 against ZZ1 are worth further investigation in future studies. With respect to its life cycle in the sensitive strain AB09V, ZZ1 proliferates efficiently, with a short latent period (9 min), a large burst size (200 PFU/ml), and a high adsorption rate. Remarkably, only less than 50 CFU/ml of the AB09V cells remained viable 30 min after AB09V cells were mixed with ZZ1 particles at a multiplicity of infection (MOI) of 10 at 37 °C. Moreover, ZZ1 exhibited the most powerful antibacterial activity at temperatures ranging from 35°C to 39°C, suggesting that the phage would be highly effective when placed inside the body at normal or near normal body temperature. In addition, ZZ1 was stable over a wide pH range (4-9) and was strongly resistant to heat. All of these features have implications for the use of this phage as a stable therapeutic agent for the treatment of *A. baumannii* infections, especially those caused by the strain most sensitive to the phage, AB09V. The differences in the antibacterial activity of ZZ1 against the strains tested will be the focus of our future research both *in vitro* and *in vivo*.

## Conclusions

This study provides information about a novel virulent *A. baumannii* phage. Our future research will examine the application of this characterized phage in treating infections by *A. baumannii* clinical isolates both *in vivo* and *in vitro*.

## Methods

### Bacterial strains and Identification

Twenty-three clinical strains of *A. baumannii* were used in this study for phage isolation and phage host investigation. All of these strains were isolated from the sputum of hospitalized patients at the Henan Province People’s Hospital in Zhengzhou, China. After obtaining the approval of the Life Science Ethics Committee of Zhengzhou University and written informed consent, sputum samples were collected for the purposes of this study. The automated system BD Phoenix (Becton Dickinson Diagnostic Systems, Sparks, MD, USA) was used on clinical samples for the identification of bacteria and for antibiotic susceptibility tests. Only 3 of the 23 strains could be lysed by ZZ1; these were lysed to varying degrees. Therefore, the 3 strains were designated AB09V, AB0901, and AB0902 in our nomenclature. The 3 strains selected for use in this study were further confirmed as *A. baumannii* using sequence information derived from their 16 S rRNA gene. Briefly, bacterial DNA was isolated as previously described [24]. The extracted DNA was used as the PCR template to amplify the 16 S ribosomal RNA coding regions. The ClustalX 2.0 program and Oligo 4.0 primer analysis software were used for universal primer design based on homology profiles among the 16 S rRNA genes of *A. baumannii* strains reported in the GenBank sequence database. The universal primers 199f (5' CTA CGG GAG AAA GCA GGG GAT 3') and 1344r (5' TTA CTA GCG ATT CCG ACT TCA 3') were used to amplify partial 16 S rRNA gene sequences. To increase the specificity of amplification and to reduce the formation of spurious byproducts, a “touchdown” PCR was performed (the annealing temperature decreased from 65 to 55°C for 20 cycles) as described previously [24]. The PCR amplicons were purified with a CONCERT Rapid PCR purification kit (Invitrogen) and were then sequenced directly with the primers.

### Bacteriophage isolation and growth

Phage isolation was conducted using the method described by Adams [25]. Several water samples (municipal sewage, fishpond water, and river water) collected from different places in Zhengzhou, China, were clarified by centrifugation (12,000 × *g* for 15 min at 4°C). One percent (v/v) of a bacterial broth culture (overnight growth) along with an equal volume of nutrient broth at double concentration was added to the cleared supernatant and incubated at 37°C overnight. The next day, after centrifugation (12,000 × *g* for 20 min at 4°C), the supernatant was filtered with a 0.45 μm SFCA Corning syringe filter (Corning Inc., Corning, NY) to remove the residual bacterial cells. An aliquot (0.2 ml) of the filtrate was mixed with 0.1 ml of an overnight culture of an *A. baumannii* strain and 2.5 ml of molten top soft nutrient agar (0.7% agar) at 47°C then overlaid on the surface of solidified base nutrient agar (1.5% agar) at 37°C. After incubation overnight at 37°C, the phage plaques were picked from the plates, and each individual plaque was re-isolated three times to ensure the purity of the phage isolate [26]. The phage titer was determined by the double-layered method [25].

Phage stocks were prepared on the most sensitive bacterial host using the soft layer plaque technique. Briefly, 10 ml of an overnight AB09V bacterial culture was concentrated to 1 ml by centrifugation (3,000 × *g* for 10 min). One hundred microliters of the concentrated culture (10^10^ CFU/ml) and 0.1 ml of the phage ZZ1 (10^7^PFU/ml) were added to 2.5 ml of molten top soft nutrient agar (0.4% agar) then overlaid on the surface of solidified base nutrient agar (1.5% agar). The plates were incubated for 6-8 h at 37°C and were used to prepare a concentrated phage suspension (10^11^PFU/ml) by eluting the top agar overlaid plates in 5 ml SM buffer. Phage stocks were stored at 4°C after filtration through 0.45-μm filters.

### Host range investigation

The host range of the phages was examined by spot tests on 23 *A. baumannii* clinical strains. A 0.1 ml aliquot of bacterial overnight broth culture (10^9^ CFU/ml) was mixed with melted 0.7% soft nutrient agar (47°C), and this mixture was poured onto 1.5% solid agar to make double layer ager plates. When the top agar hardened, phage stock (5 μl) from a dilution series was spotted on each plate with different bacterial strains. The plates were incubated at 37°C. When clearing zones were observed, the antibacterial activity of the phages against each bacterial host was assessed based on the minimum phage concentration required to form a completely transparent zone.

### Investigation of ZZ1 antimicrobial activity against AB09V at different temperatures

The antibacterial activity of ZZ1 against *A. baumannii* AB09V was evaluated by serial dilution spot testing at different temperatures. Phage stock (5 μl) from a dilution series was spotted onto a lawn of AB09V in top agar. The plates were examined for cell lysis after overnight incubations at 25°C, 30°C, 35°C, 37°C, 39°C, 40°C, and 42°C. The optimal antibacterial temperature was determined by comparing the minimum phage concentration required to form a completely transparent zone.

### Phage adsorption and growth curve

An overnight culture of strain AB09V (1 ml) was inoculated into fresh medium (100 ml) and incubated with shaking at 37°C for approximately 1 h to yield a cell density of approximately 7.0 × 10^7^ CFU/ml (at an OD_600_ of 0.15). A 1 ml sample of a nutrient broth suspension of the phage ZZ1 at an approximate MOI of 10 was added to this culture. Samples were periodically withdrawn and immediately chilled while being further diluted to measure total phage activity (including infected bacterial cells and free phages) by the double-layered-agar plate technique. Bacterial viable counts were determined before the bacteria were mixed with the phage and were assessed periodically. Burst size was estimated from triplicate experiments using the equation described by Jiang et al. [27]. Each experiment was performed three times, and the results are reported as the mean of three observations ± standard deviation (SD).

### Stability

Resistance to different pH values at 37°C was determined according to the methods described by Verma et al. [28]. The pH of the nutrient broth was adjusted with either 1 M HCl or 1 M NaOH to obtain a pH within the range of 2–11. A total of 100 μl of bacteriophage suspension (4.7 × 10^11^ PFU/ml) was inoculated into 10 ml of pH-adjusted medium. After incubation for 1 h at 37°C, the surviving phages were diluted and counted immediately using the soft agar overlay method at 37°C. Moreover, according to the methods described by Capra et al. [29], the stability of ZZ1 at various temperatures (50°C, 60°C, 70°C, and 80°C) was checked by incubating the phage (3.2 × 10^10^ PFU/ml) at the indicated temperature for 1 h at pH 7.0 in nutrient broth; the surviving phages were then counted using the soft agar overlay method at 37°C.

### Morphology of phage and its host strain

AB09V cells were infected with ZZ1 during the exponential growth phase (OD_600_ = 0.35) at an MOI of approximately 100 and incubated at 37°C for 5 min in nutrient broth medium. The mixture was fixed with 1% glutaraldehyde at 0°C for 60 min and then centrifuged (4500 × *g*, 3 min). The phage-cell complexes from the pellets were placed on 300-mesh copper grids coated with carbon film and then stained with phosphotungstic acid (2% w/v, pH 7.2) for 30 s. After drying, the preparation was examined by a transmission electron microscope.

### Genome sequencing and analysis

The nucleic acid of phage ZZ1 was isolated as previously described [20]. Purified nucleic acid was used to determine susceptibility to DNase, RNase, and restriction enzymes and was then sent to Zhejiang California International NanoSystems Institute (Hangzhou, China) for commercial sequencing. The whole genome sequence, with a total length of 166,682 bp, was obtained using the Illumina Solexa Sequencing platform (Illumina, San Diego, USA) and the Swift analysis tool (http://swiftng.sourceforge.net) [30]. The genome sequence was analyzed with the NCBI BlastX bioinformatics tool (http://blast.ncbi.nlm.nih.gov/Blast.cgi) for nucleotide analysis, and the NCBI ORF finder (http://www.ncbi.nlm.nih.gov/projects/gorf/) was used to identify ORFs, which were limited to those encoding proteins of greater than or equal to 50 amino acids. Homology assignments between genes from other phages and predicted ORFs of phage ZZ1 were based on amino acid sequence alignment searches (BlastP, http://blast.ncbi.nlm.nih.gov/Blast.cgi).

### Nucleotide sequence accession number

The genome sequence, with a total length of 166,682 bp, for phage ZZ1 described in this work was submitted to GenBank and was assigned the accession number [GenBank: HQ698922].

## Competing interests

The authors declare that they have no competing interests.

## Authors' contributions

JJ conceived of the study and designed all the experiments and drafted the manuscript; ZJL, SWW, and DHH performed all phage-related experiments; SMW, YYM, and JW analyzed the clinical bacteria strains; FL and XDC participated in the TEM investigation; YHL, GXL, and XTW analyzed the phage genome. GQZ and ZQW participated in the design of the study and coordination and helped to draft the manuscript. All authors read and approved the final manuscript.
